# Supplementary material for: DNA transposons mediate duplications via transposition-independent and -dependent mechanisms in metazoans
Source: Nat Commun. 2021 Jul 13;12:4280. doi: 10.1038/s41467-021-24585-9 (PMC8277862; doi:10.1038/s41467-021-24585-9)
Supplement: Supplementary file 1 — Supplementary Information [file 41467_2021_24585_MOESM1_ESM.docx]

**Supplementary Information for**

DNA transposons mediate duplications via transposition-independent and -dependent mechanisms in metazoans

Shengjun Tan,^#^ Huijing Ma,^#^ Jinbo Wang,^#^ Man Wang,^#^ Mengxia Wang, Haodong Yin, Yaqiong Zhang, Xinying Zhang, Jieyu Shen, Danyang Wang, Graham L. Banes, Zhihua Zhang, Jianmin Wu, Xun Huang, Hua Chen, Siqin Ge, Chun-Long Chen,^*^ Yong E. Zhang^*^

#These authors contributed equally: Shengjun Tan, Huijing Ma, Jinbo Wang, Man Wang.

*Correspondence should be addressed to Chun-Long Chen ([chunlong.chen@curie.fr](mailto:chunlong.chen@curie.fr)) or Yong E. Zhang ([zhangyong@ioz.ac.cn](mailto:zhangyong@ioz.ac.cn)).

**Supplementary Figures**


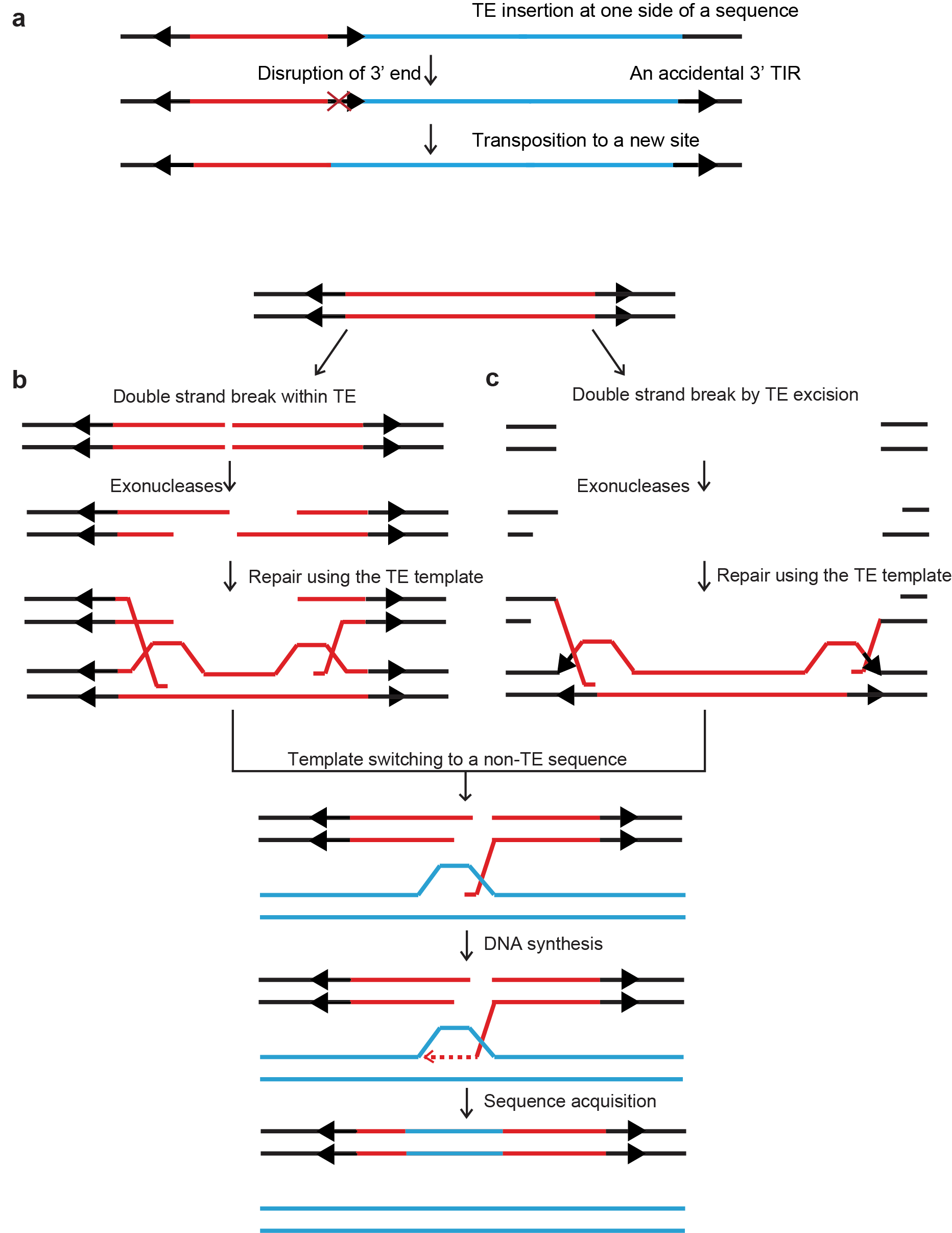


**Supplementary Figure 1. Known models on the mechanism through which TIR TEs capture sequences. a** End bypass model. **b-c** Gap-filling model. Double-strand breaks (DSBs) occur within TEs due to fragile sites (**b**) or excision of active TEs (**c**), and this occurrence was followed by 5’-end resection by exonucleases and gap repair. The strand under repair might switch to a non-TE sequence. Internal TE sequences and fillers are marked in red and light blue, respectively. TIRs are shown by black arrows.


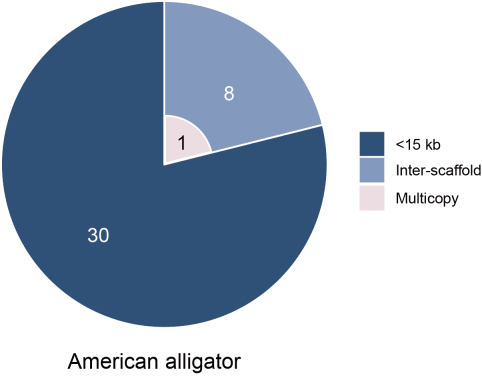


**Supplementary Figure 2. Distribution of Pack-TIRs classified by distance from their parental copies in American alligators.** The figure convention follows that of Fig. 2a.


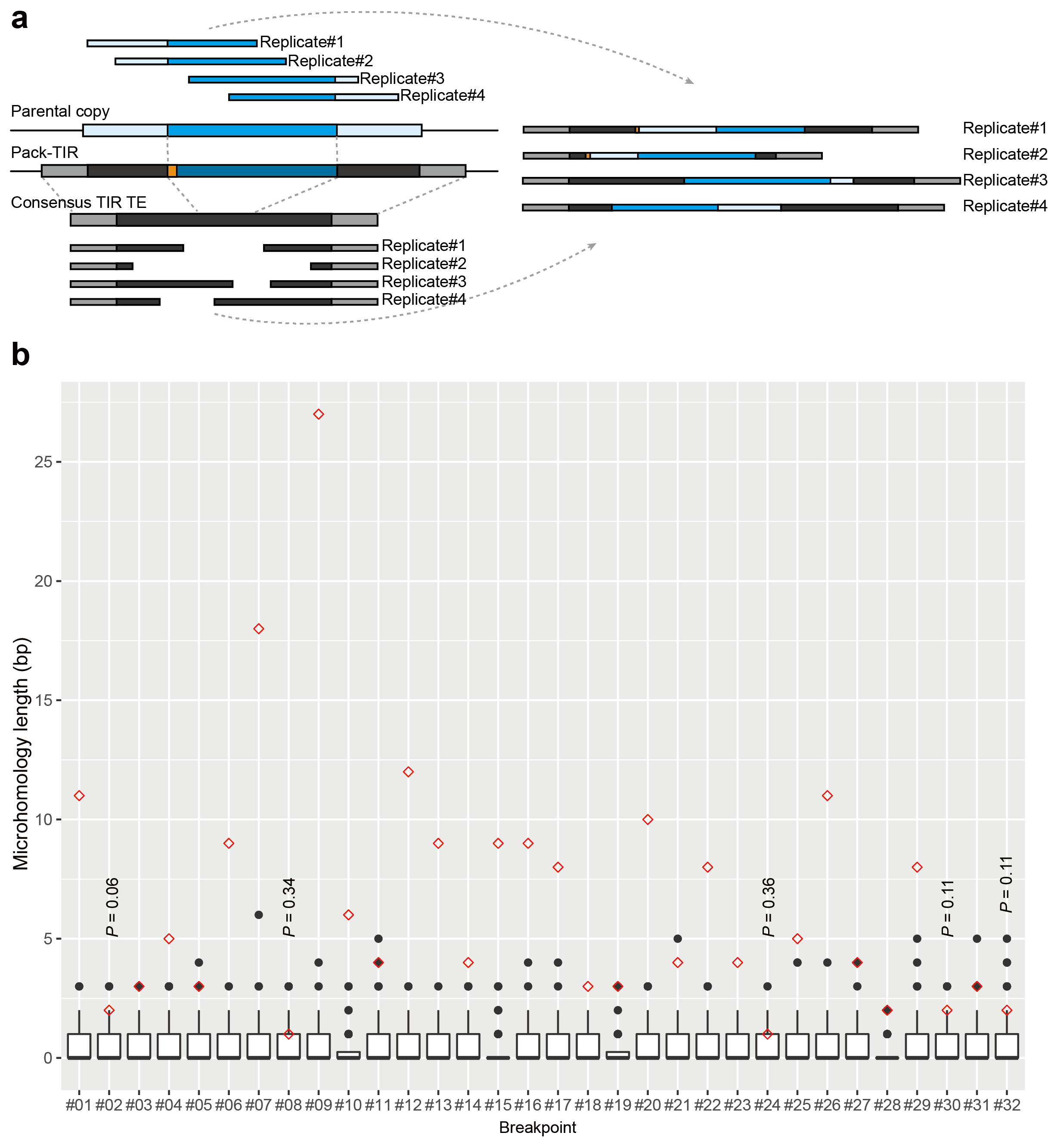


**Supplementary Figure 3. Distribution of the microhomology length across 100 simulations for 32 breakpoints. a** Simulation process testing the length of the microhomology at the left breakpoint. The figure convention follows that of Fig. 2e with the exception that the flanking regions of the parental copy are shown in light blue. The parental copy and its flanking regions (50% length of the parental copy) were used as the template. Switching points in the consensus TIR TE and parental copy were randomly selected. After constructing 100 pseudo Pack-TIRs, we recorded the size of the microhomology (marked in orange) at the left breakpoint. Four replicates are shown as examples, microhomology was detected for two replicates, and none of these was as long as the observed microhomology. **b** The distribution of random samples (*n* = 100 samples) is shown as a boxplot with the middle box, the upper and lower whiskers, and the black line representing the interquartile range, the extreme values, and the median, respectively. The red diamonds indicate the observed values. *P*-values are calculated based on random sampling, and only *P*-values not lower than 0.05 are shown.


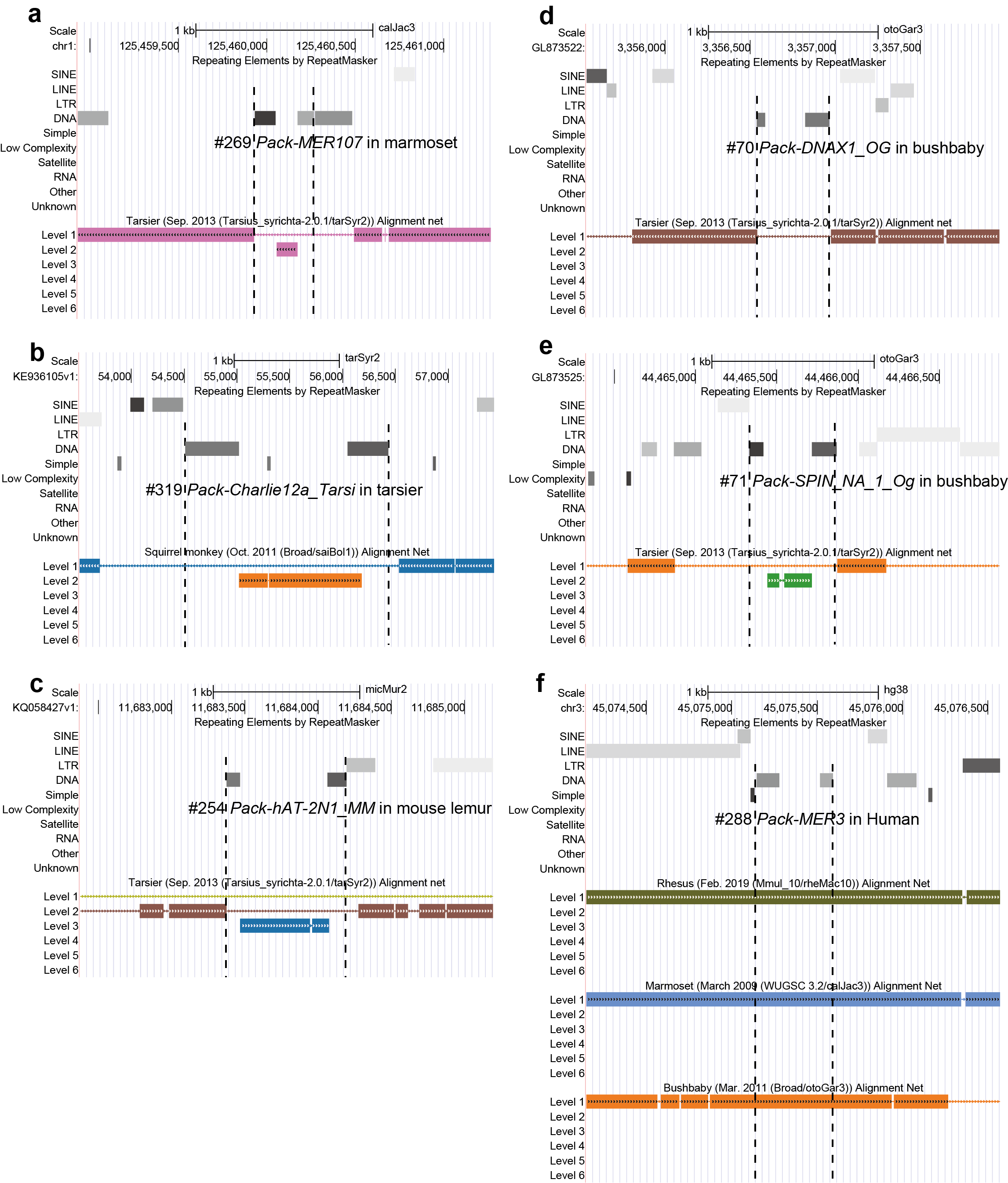


**Supplementary Figure 4. Six Pack-TIRs completely absent in the outgroup species or shared by primates. a** Pack-TIR #269 together with the flanking 8-bp and 242-bp sequences was absent in tarsiers. **b** Pack-TIR #319 together with the flanking 807-bp and 103-bp sequences was absent in squirrel monkeys. **c** Pack-TIR #254 together with the flanking 1-bp and 80-bp sequences was absent in tarsiers. **d** Pack-TIR #70 together with the flanking 0-bp and 8-bp sequences was absent in tarsiers. **e** Pack-TIR #71 together with the flanking 457-bp and 1-bp sequences was absent in tarsiers. **f** Pack-TIR #288 were present in 16 out of 18 primates. These snapshots were from the UCSC genome browser, which uses Net Track to represent genome-wide synteny. Upper-level Net is more likely orthologous, and lower-level Net might represent one-way paralogous mapping. In each case, we manually checked whether the Pack-TIR and its flanking 1000-bp sequences were shared across primates. Because outgroup species generally show consistent patterns, we showed the net track belonging to one of the most closely phylogenetically related species for all the cases with the exception of the last case (Panel f), for which we showed tracks from multiple phylogenetically representative species.


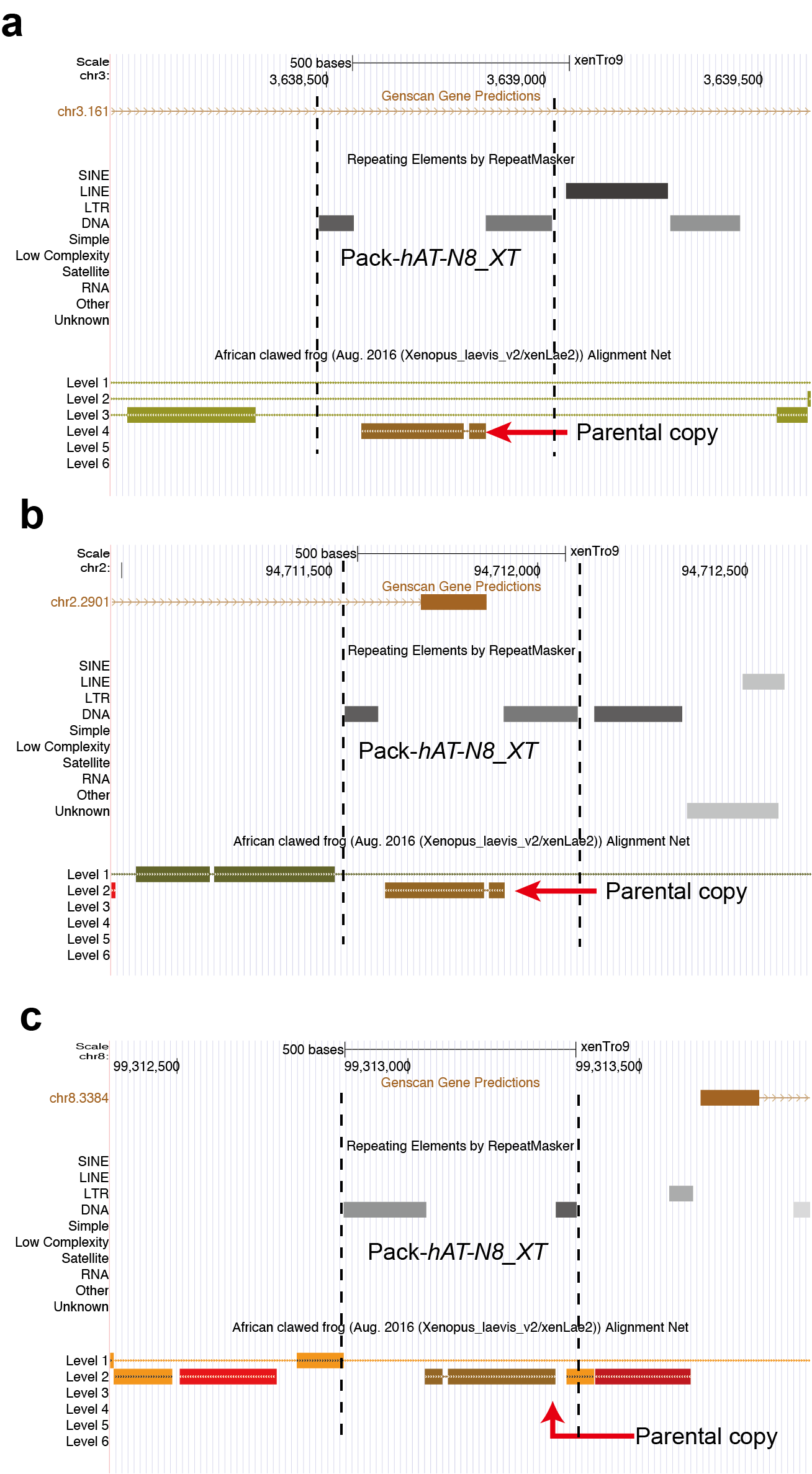


**Supplementary Figure 5. The multicopy Pack-TIRs in western clawed frogs are absent in the outgroup, African clawed frogs.** The snapshots for each copy of *Pack-hAT-N8_XT* (**a**, **b** and **c**, respectively) are from the frog genome (*Xenopus tropicalis*, xenTro9) in the UCSC genome browser. In addition to the net track, Genscan gene annotations and repeat annotations are also shown. For these Pack-TIRs, the neighboring region could be shared by the outgroup (*e.g.*, the left flanking region demonstrated by Level 1 Net in Panel b). In contrast, Pack-TIRs are always absent in Level 1 Net, although paralogous mappings to the parental copy exist in Level 2.


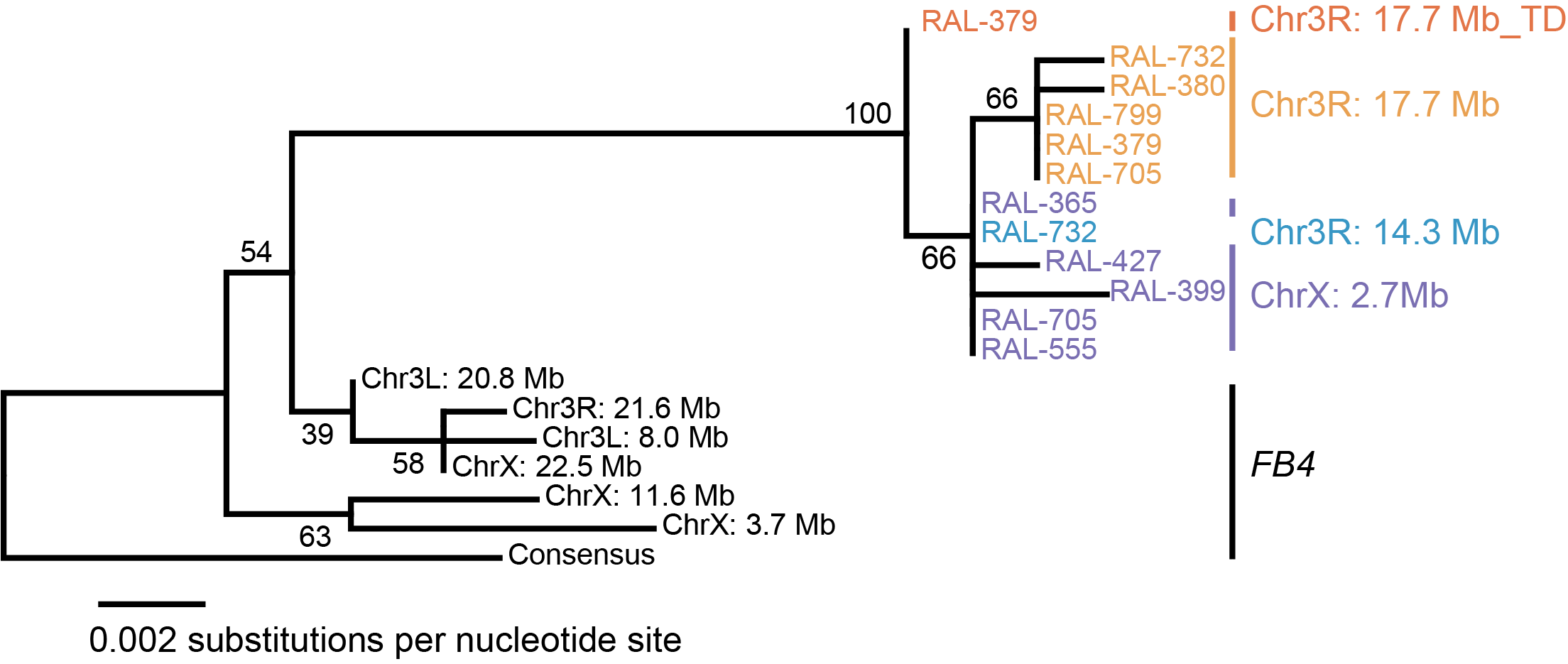


**Supplementary Figure 6. Phylogenetic tree of *FB4*-derived sequences in *Ssk-FB4s*.** The consensus *FB4* from RepBase was used as the outgroup. Because *FB4s* themselves are repetitive with smaller repeats nested in larger repeats, the bootstrap score is generally lower for this tree compared with the *Ssk*-derived sequence-based tree in Fig. 3c. Different *Ssk-FB4s* are color-coded as in Fig. 3c.


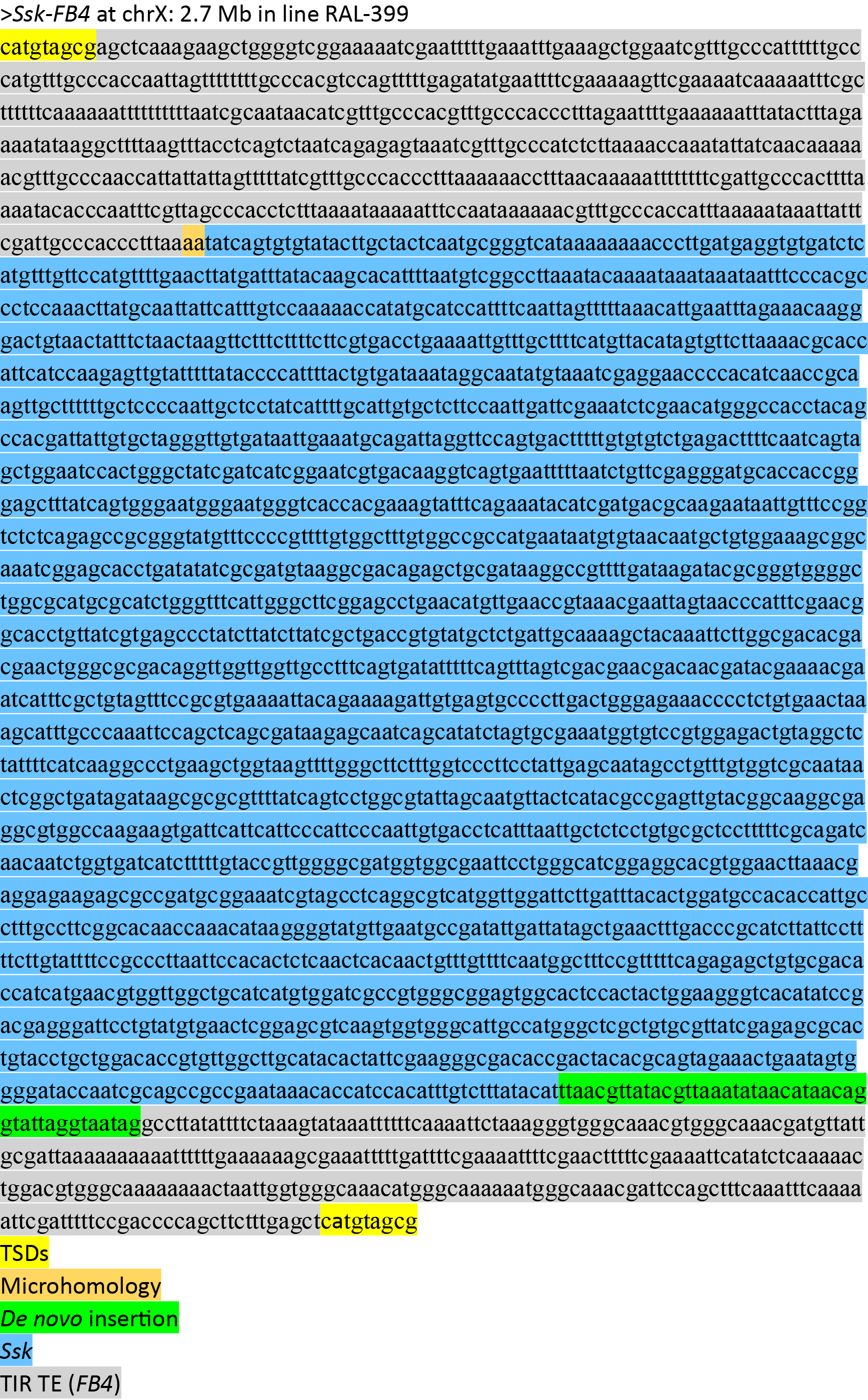


**Supplementary Figure 7. Sequence of *Ssk-FB4*.** The color codes are indicated.


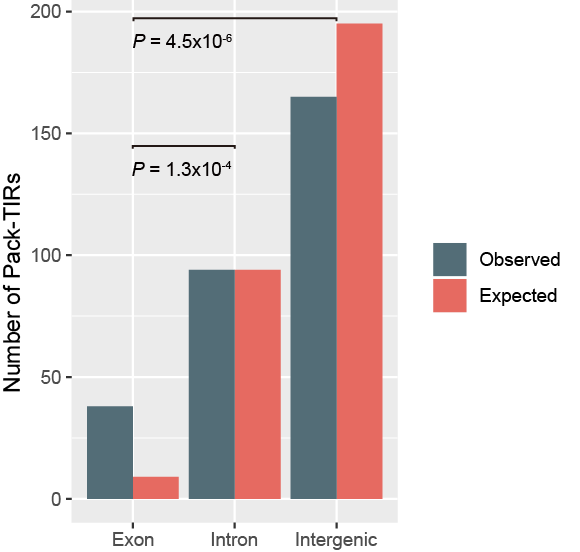


**Supplementary Figure 8. Distribution of functional types of parental sequences across all species.** The figure convention follows that of Fig. 4b. The one-sided Wilcoxon rank sum test was used to calculate the *P*-value.


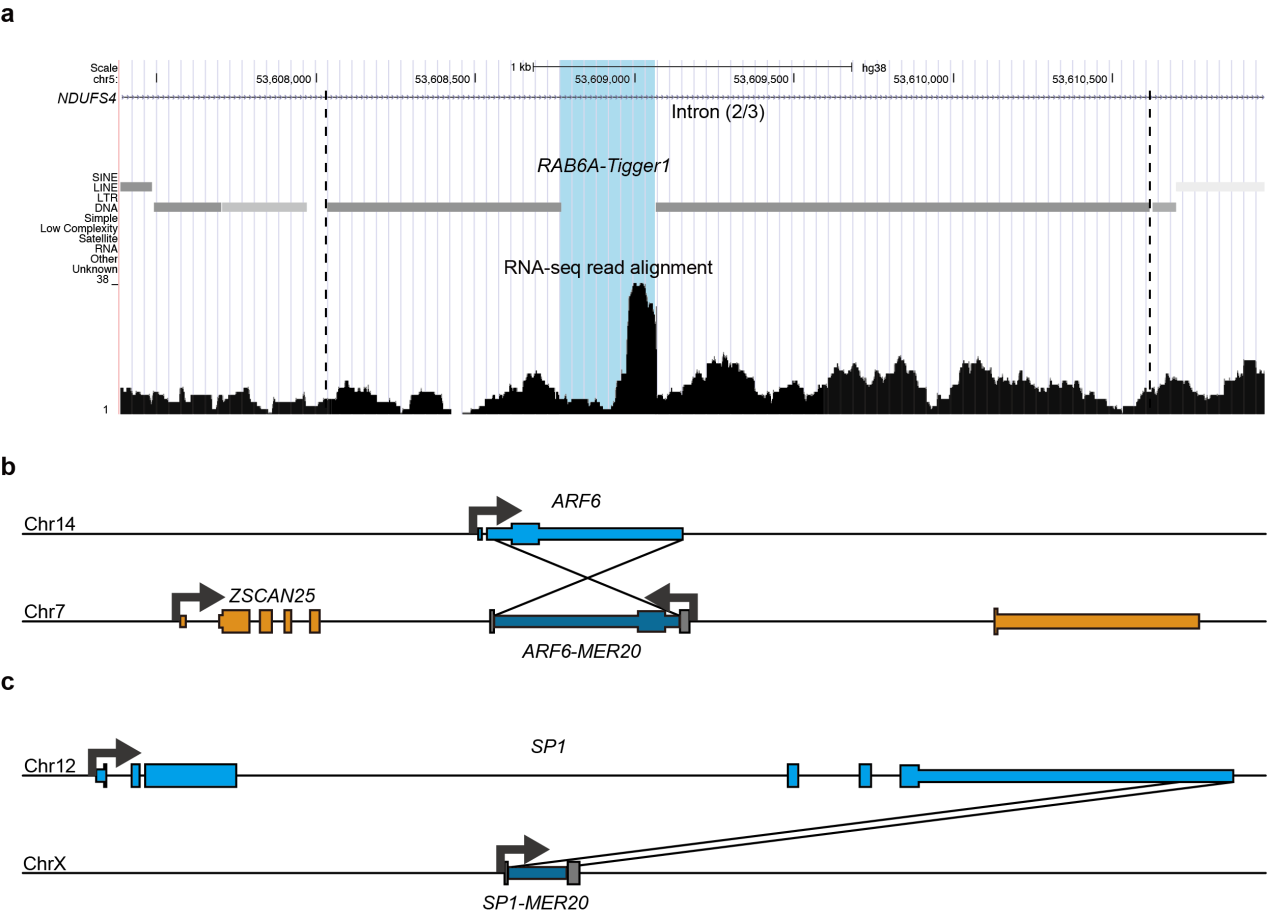


**Supplementary Figure 9. Three Pack-TIR examples in humans.** **a** A UCSC snapshot of the human genome (hg38) at the second intron of *NDUFS4*. RefSeq gene annotation, repeat annotation and RNA-seq alignment tracks are shown. *RAB6A-Tigger1* was inserted in the second intron of *NDUFS4* and the uniquely mapped stranded RNA-seq reads suggested intron retention. The *RAB6A*-derived region is shaded in light blue. **b** *ARF6* duplication. *ARF6* is a known retrogene. Because the duplication only covers its second exon and does not involve any intron, whether a recurrent retroposition or *MER20* mediated its origination remains uncertain. **c** Part of *SP1* was captured by *MER20* into an intergenic region. The figure convention in Panels b and c follows that of Fig. 4d with the exception that TIR TEs are shown in the name of Pack-TIRs.

**
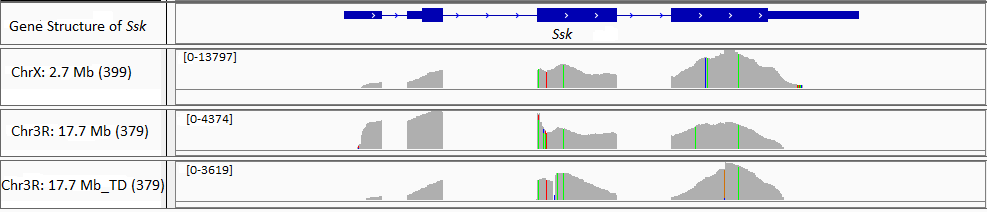
**

**Supplementary Figure 10. Gene structure of S*sk-FB4s*.** RNA-seq read mapping results in IGV show the gene structure of *Ssk-FB4s* at chrX: 2.7 Mb in the RAL-399 line and chr3R: 17.7 Mb and the tandem duplicated locus in the RAL-379 line. The thinner blue boxes, the thicker boxes and the intervening lines represent the UTR, coding exons and introns of *Ssk*, respectively. The number (*e.g.*, 0-13797) indicates the range of the RNA-seq read depth. The colored lines represent the nucleotide changes between *Ssk-FB4s* and *Ssk*.


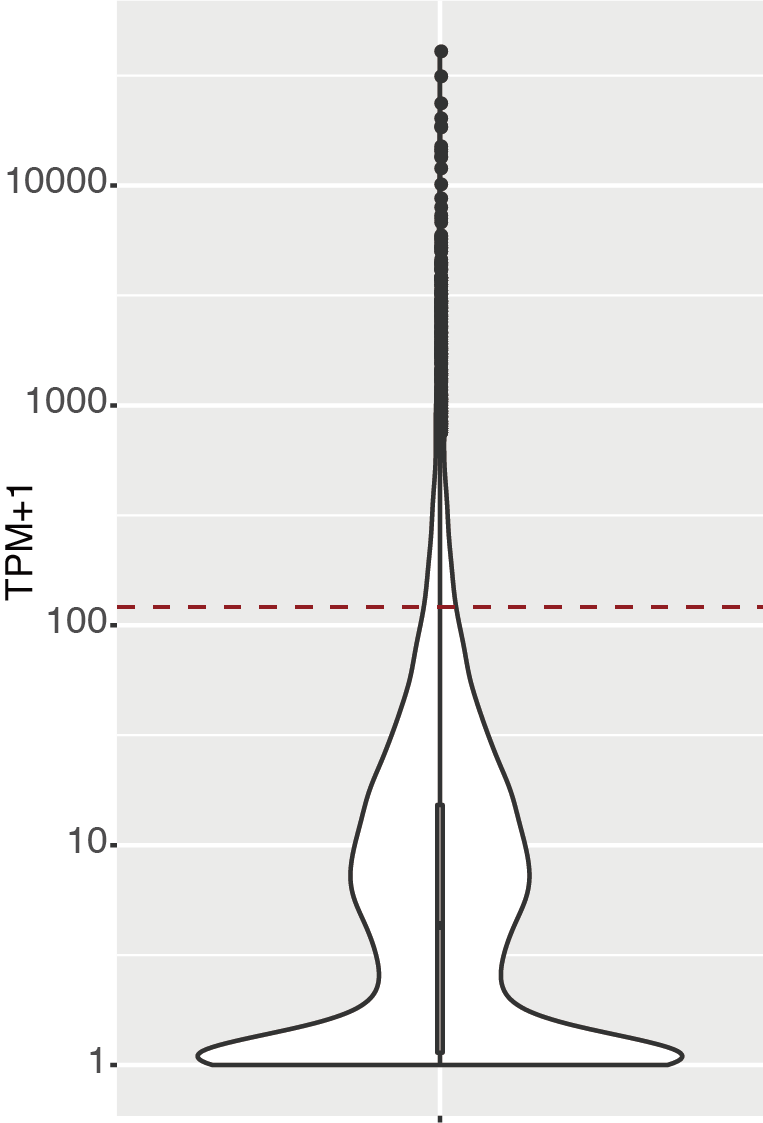


**Supplementary Figure 11. Transcription level of all protein-coding genes (*n* = 13,933 samples) in the midgut.** For all protein-coding genes, the distribution of their median expression level (TPM+1) across six lines is shown as a log_10_ scaled violin plot. See Fig. 2f for a definition of a violin plot. The red dashed line marks the median expression of *Ssk-FB4s*, *i.e.*, 120.


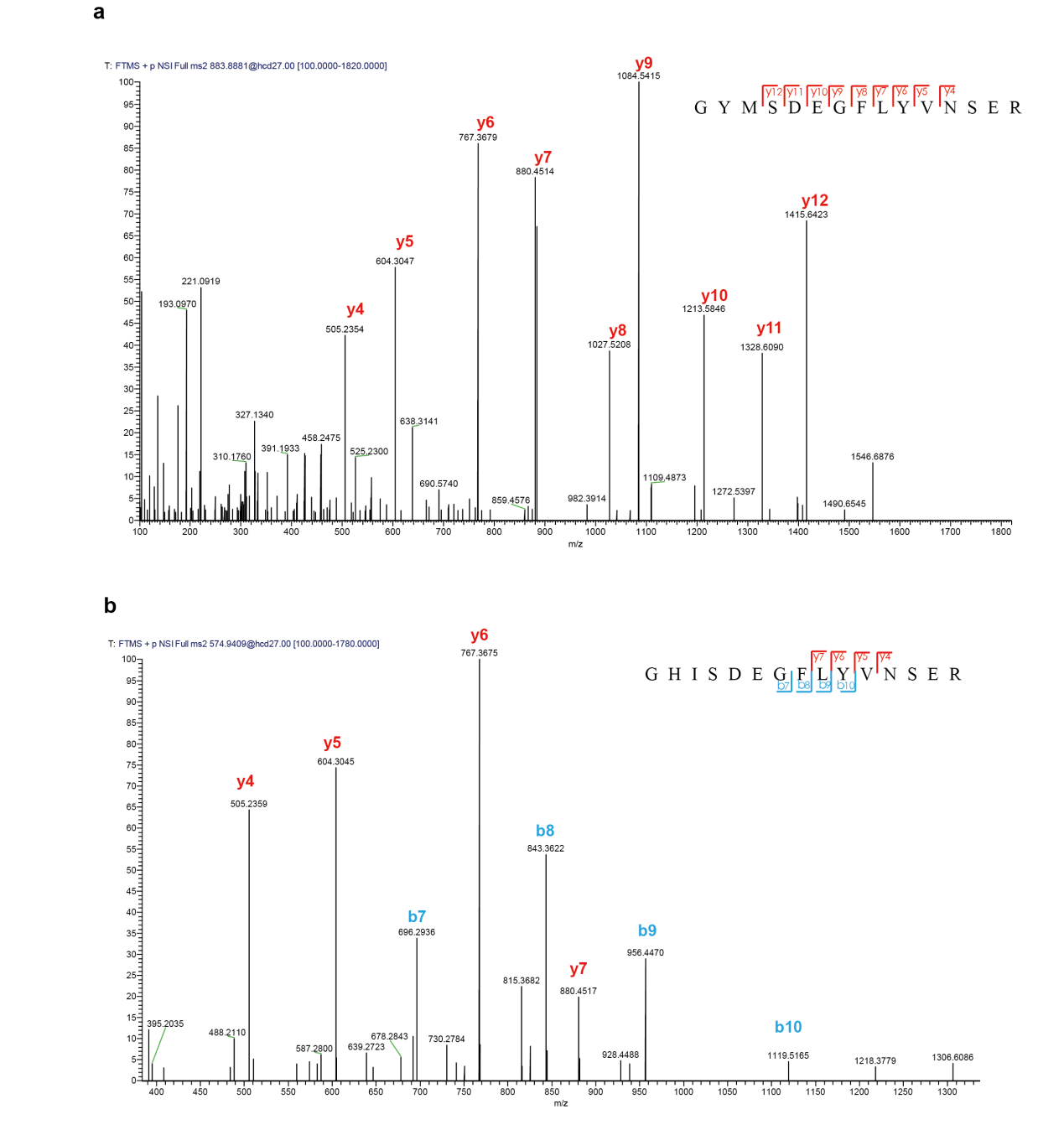

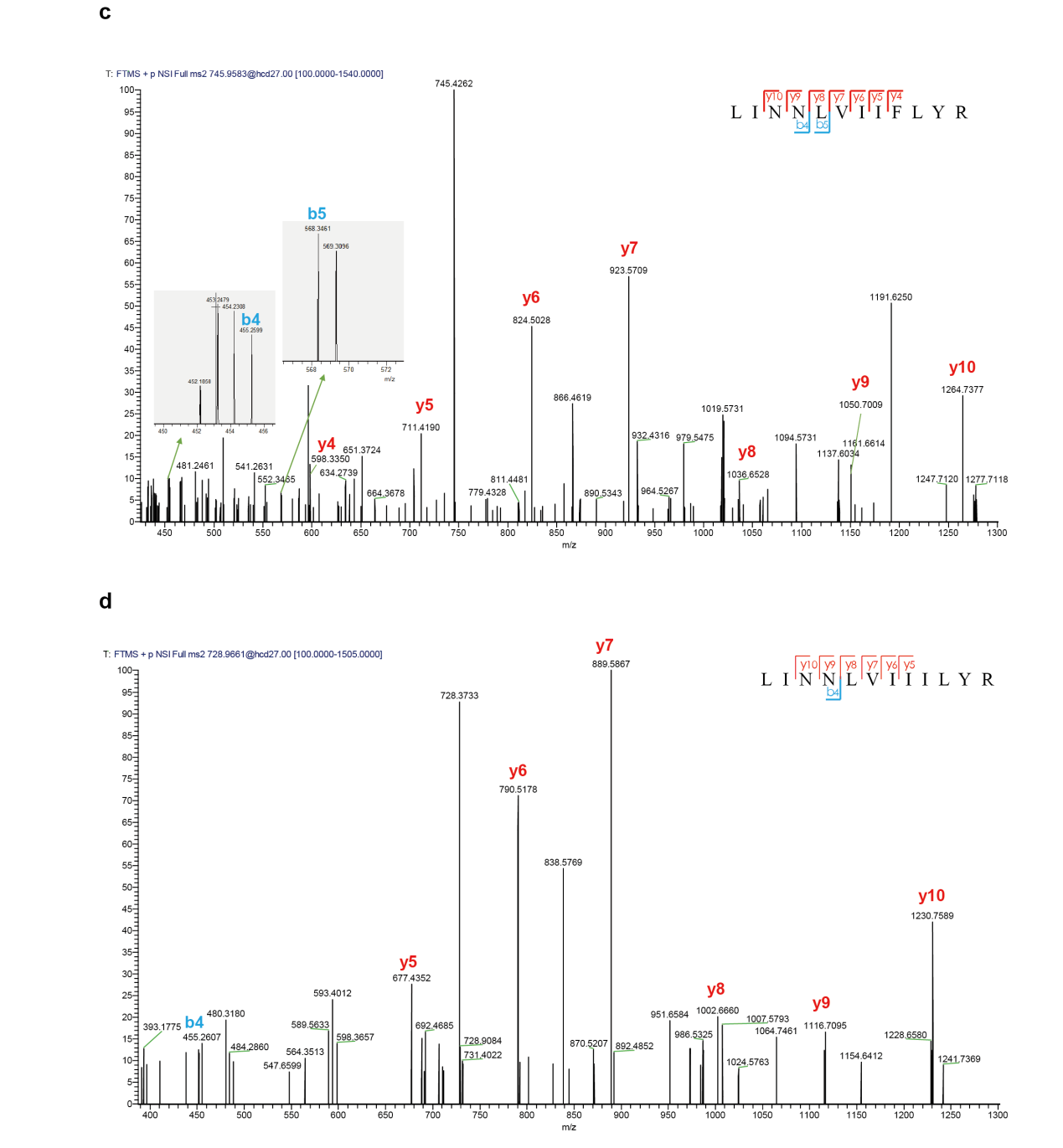


**Supplementary Figure 12. High-quality peptide spectrum identified by PRM-MS assay. a** GYMSDEGFLYVNSER is the unique peptide of Ssk in the RAL-399 and RAL-427 lines. **b** GHISDEGFLYVNSER is the unique peptide of Ssk-FB4 (chrX: 2.7 Mb) in the RAL-399 and RAL-427 lines. **c** LINNLVIIFLYR is the unique peptide of Ssk-FB4 (chrX: 2.7 Mb) in the RAL-399 line and Ssk-FB4 (chr3R: 17.7 Mb_TD) in the RAL-379 line. **d** LINNLVIIILYR is the unique peptide of Ssk-FB4 (chr3R: 17.7 Mb) in the RAL-379 line. In Panels a-d, the N- and C-terminal collision-induced dissociation fragment ions are shown by the b and y series, respectively. Taking Panel d as an example, high-quality ions (y5-y10, b4) are shown as individual peaks, and y10 and b4 are interpreted as NNLVIIILYR and LINN, respectively. Similar to y10, y5 to y9 are interpreted accordingly. Altogether, a peptide, *i.e.*, LINNLVIIILYR could be jointly called.


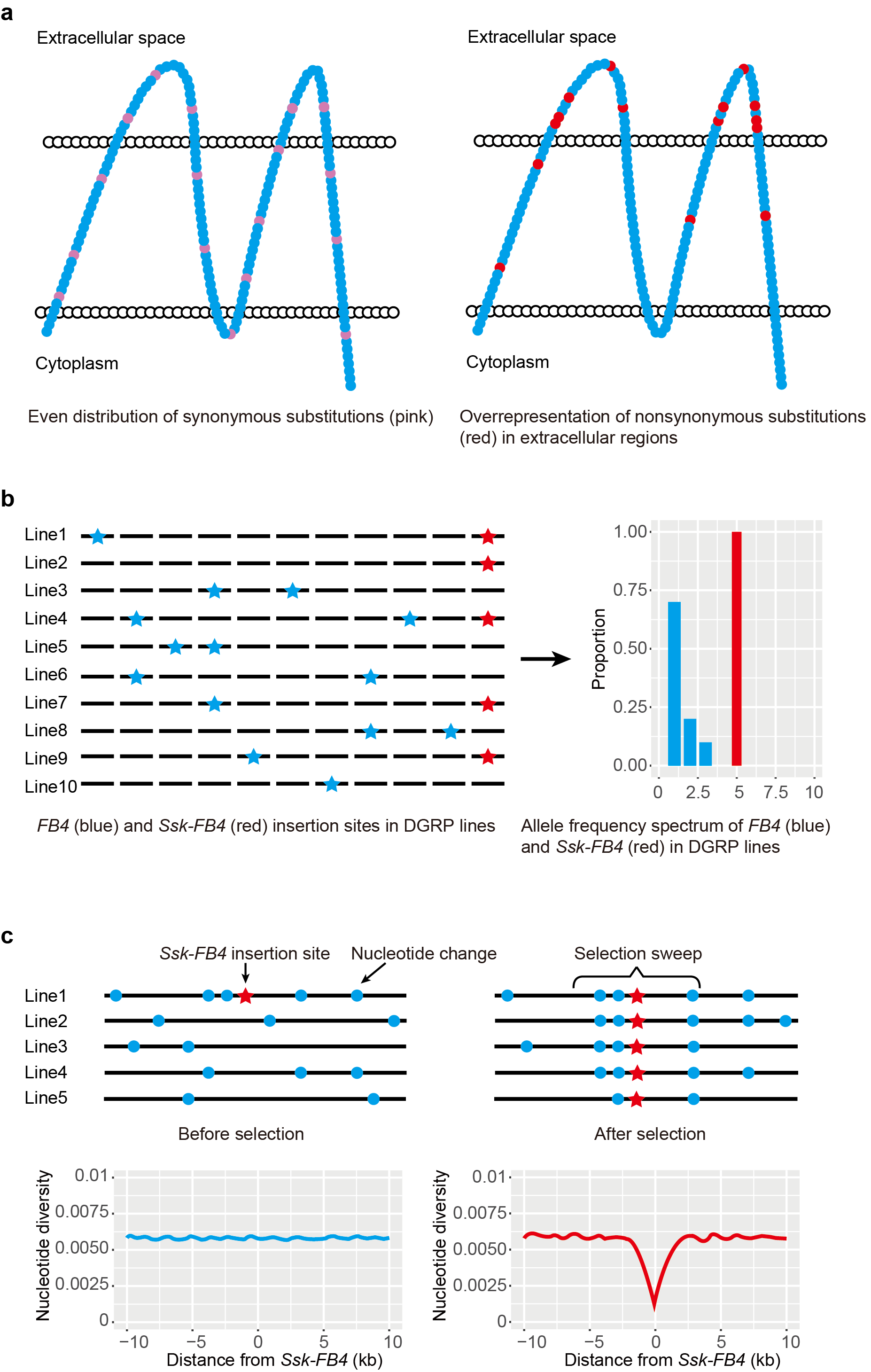


**Supplementary Figure 13. Illustrative cartoons of signatures associated with positive selection acting on *Ssk-FB4s*. a** Nonsynonymous substitutions are enriched in one particular functional region (*e.g.*, extracellular region) relative to the even distribution of synonymous substitutions. **b** Allele frequency distribution of *FB4s* and *Ssk-FB4*. In the left panel, blue and red stars represent *FB4s,* and one *Ssk-FB4* insertion across lines, respectively. The frequency spectra of these mutations are summarized in the right panel with X and Y axis referring to the count of lines and proportion of the corresponding frequency groups. **c** Mutation accumulation before and after selection. In the top panel, the red star represents *Ssk-FB4* and blue dots represent flanking mutations before (left) and after (right) selection. The bottom panel shows the corresponding nucleotide diversity across sliding windows. Under positive selection, *Ssk-FB4* will rapidly increase in frequency, and nearby linked alleles hitchhike along with it, leading to a decrease in the nucleotide diversity. This is particularly pronounced for the closely linked region due to a lack of recombination. We therefore selected synonymous substitutions with a similar allele frequency and a similar recombination rate as the neutral control to test the significance of the decrease (see also Methods).


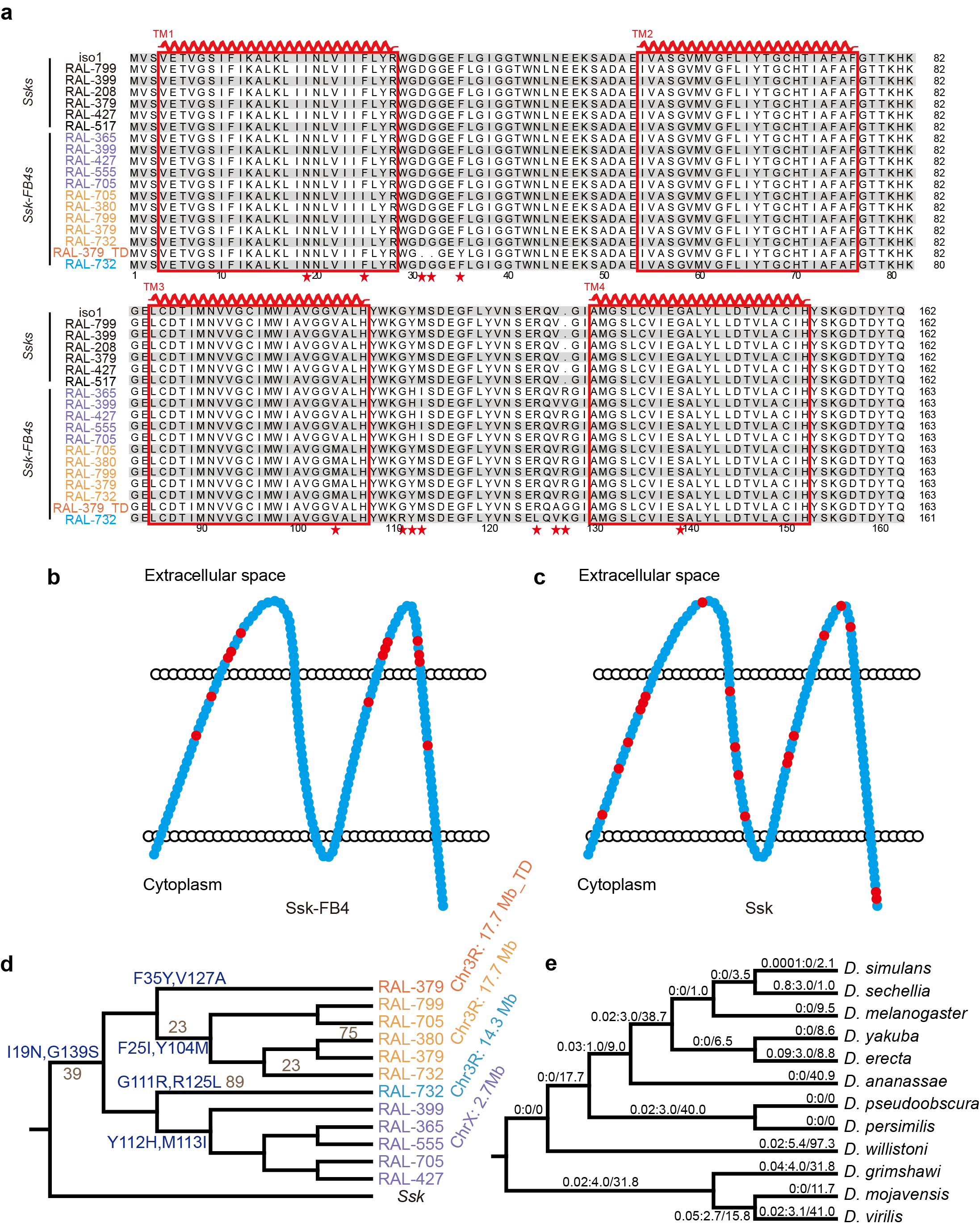


**Supplementary Figure 14. Nonsynonymous and synonymous changes in *Ssk-FB4s*. a** Alignment of *Ssk-FB4s* and *Ssks.* The red stars indicate the amino acid changes in S*sk-FB4s*. Four transmembrane (TM) domains are framed. The numbers refer to individual DGRP lines, whereas “iso1” refers to the reference line. **b-c** Schematic show of the Ssk-FB4 protein (**b**) or Ssk protein (**c**). The red dots represent amino acid changes among different domains. **d** Phylogenetic tree of *Ssk-FB4s* with nonsynonymous (blue) and synonymous (gray) SNPs labeled on the branches. The 23^rd^ codon had a back mutation in *Ssk-FB4* at chr3R: 17.7 Mb in line 732. **e** Phylogenetic tree of the *Drosophila* genus with *K_a_/K_s_* labeled on each branch as “*K_a_/K_s_* value: number of nonsynonymous substitutions/number of synonymous substitutions”.


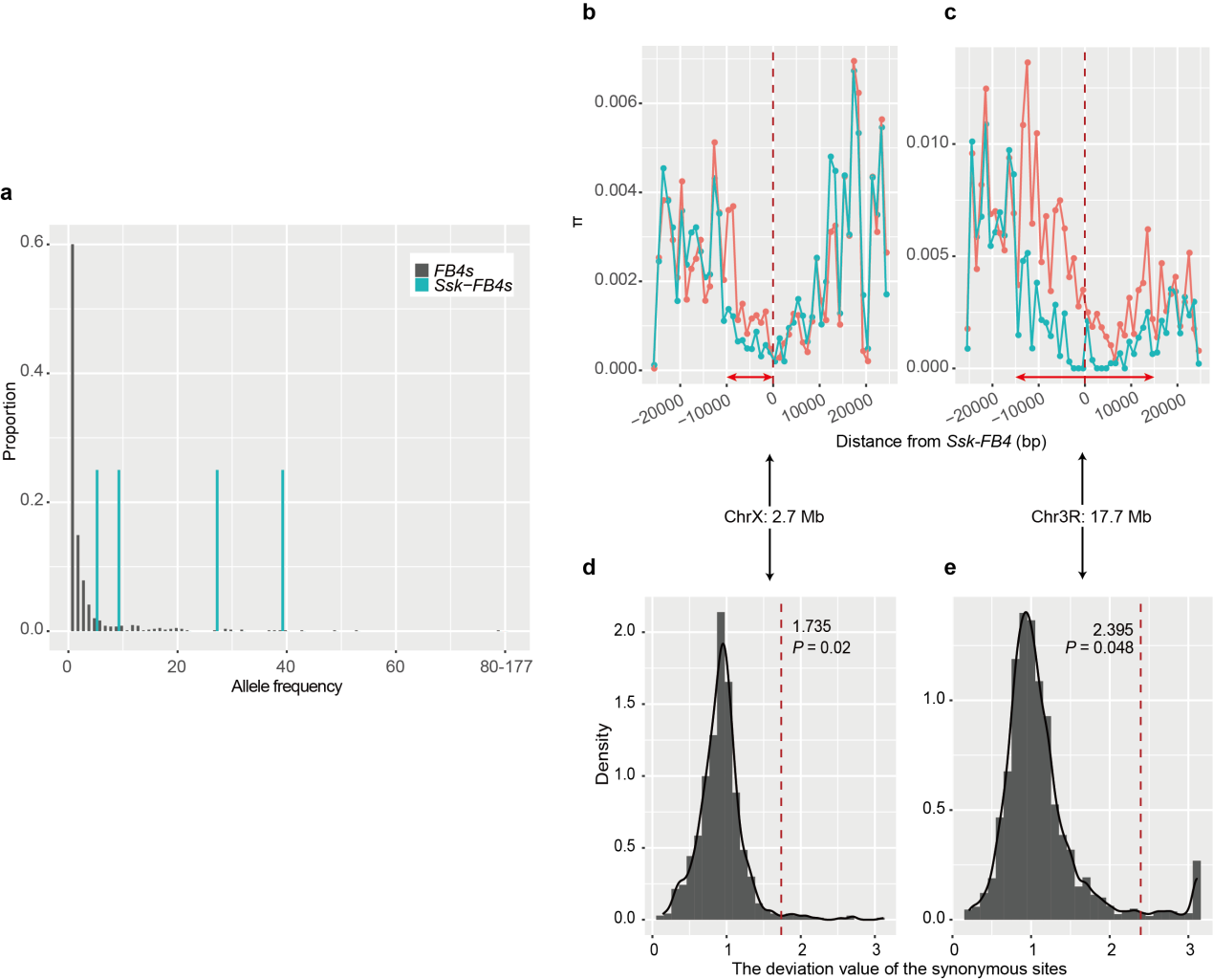


**Supplementary Figure 15. Signature of positive selection acting on S*sk-FB4s*. a** Allele frequency distribution of *FB4s* and *Ssk-FB4s* in 177 DGRP lines. This panel is similar to Fig. 5D with the exception that a different frequency dataset of TEs was used (Methods). **b-c** Sliding window of the nucleotide diversity (π) across the 50-kb region flanking S*sk-FB4* at chrX: 2.7 Mb (**b**) or chr3R: 17.7 Mb (**c**). The dark red dashed line marks the position of S*sk-FB4s*. The window size and step size were 1 kb and 500 bp, respectively. The green line refers to lines with S*sk-FB4s*, whereas the light red line refers to a comparable random sample including the same number of lines without S*sk-FB4s*. The red lines with arrows mark the 5’ 10-kb region of *Ssk-FB4* at chrX: 2.7 Mb and that of the 30-kb region of *Ssk-FB4* at chr3R: 17.7 Mb, which show lower π and are thus potentially subject to selection sweep. **d-e** Probability density distribution of the ratio (π_without_ *_Ssk-FB4_*/π_with_ *_Ssk-FB4_*) of synonymous single nucleotide polymorphisms (SNPs) on chrX (**d**) or chr3R (**e**), which have a similar allele frequency and recombination rate as the corresponding S*sk-FB4* copy (Methods). The red dashed lines indicate the ratio (π_without_ *_Ssk-FB4_*/π_with_ *_Ssk-FB4_*) of the 5’ 10-kb region at chrX: 2.7 Mb and that of the 30-kb region at chr3R: 17.7 Mb. *P*-values are calculated based on random sampling (see also Methods). Note: the 5’-only sweeping and smaller window size of X-linked *Ssk-FB4* could be due to the higher local recombination rate compared with that of the 3R-linked copy (2.6 cM/Mb *vs.* 1.6 cM/Mb, data from ^1^).


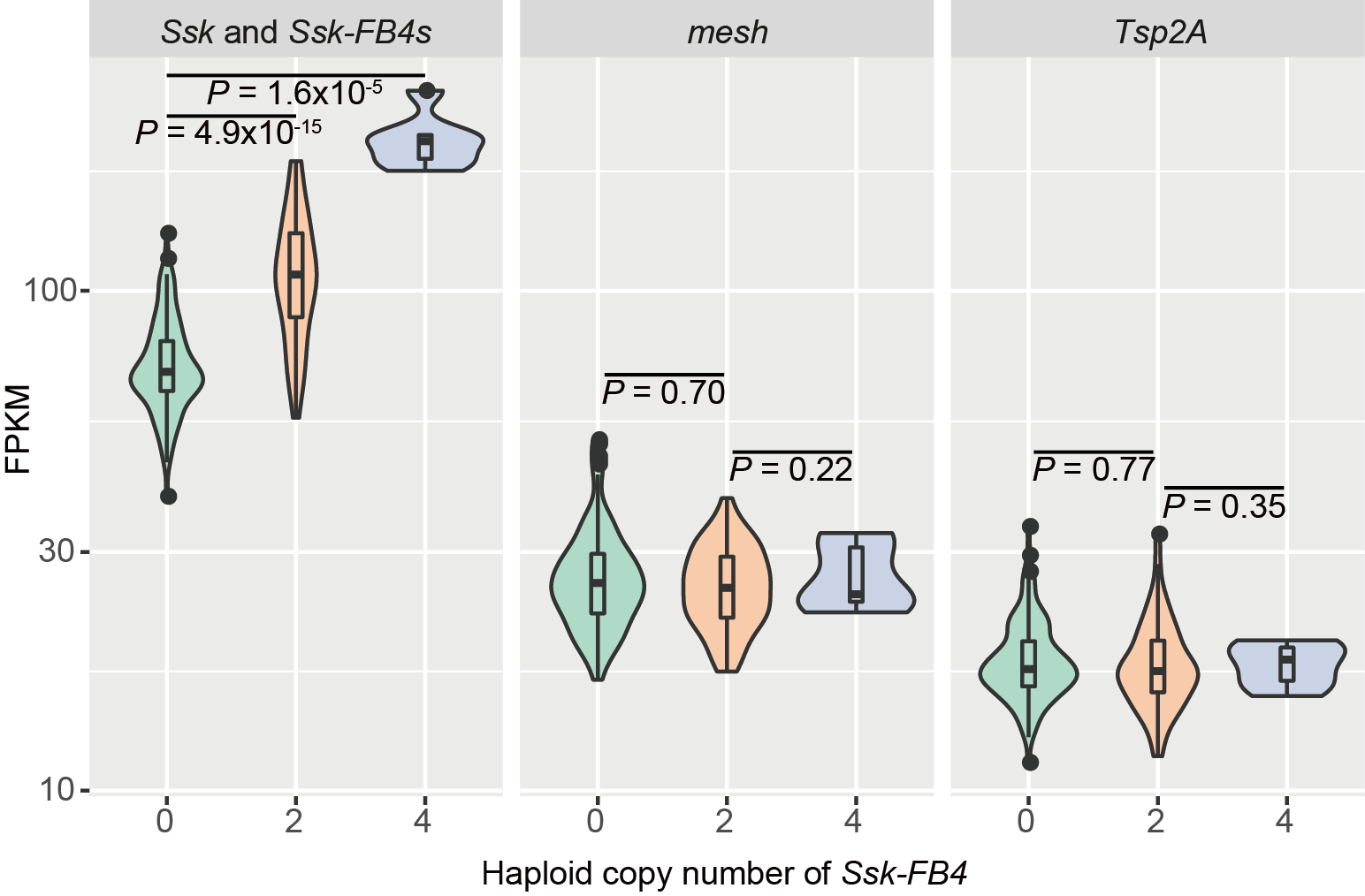


**Supplementary Figure 16. Expression of *Ssk*, *Ssk-FB4s*, *mesh* and *Tsp2A* in the whole body across 200 lines with different copy numbers of *Ssk-FB4s*.** The figure convention follows that of Fig. 5e. The distribution is shown as a violin plot as in Fig. 2f. We divided lines into three groups according to the copy number of *Ssk-FB4s*: 0 (*n* = 124 samples), 2 (*n* = 54 samples), and 4 (*n* = 7 samples). The one-sided Wilcoxon rank sum test was performed.


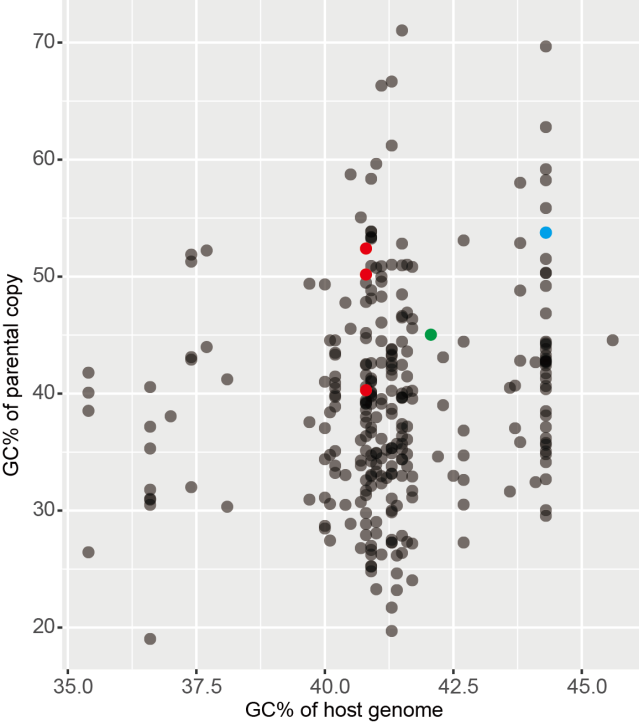


**Supplementary Figure 17. GC content of parental copies and the corresponding host genome.** A total of 282 unique Pack-TIRs across 56 species are presented. The five colored dots represent multicopy Pack-TIRs in western clawed frogs (red), and American alligators (blue) and *Ssk-FB4s* in fruit flies (green). Four out of five cases showed a higher GC% relative to the genomic background.


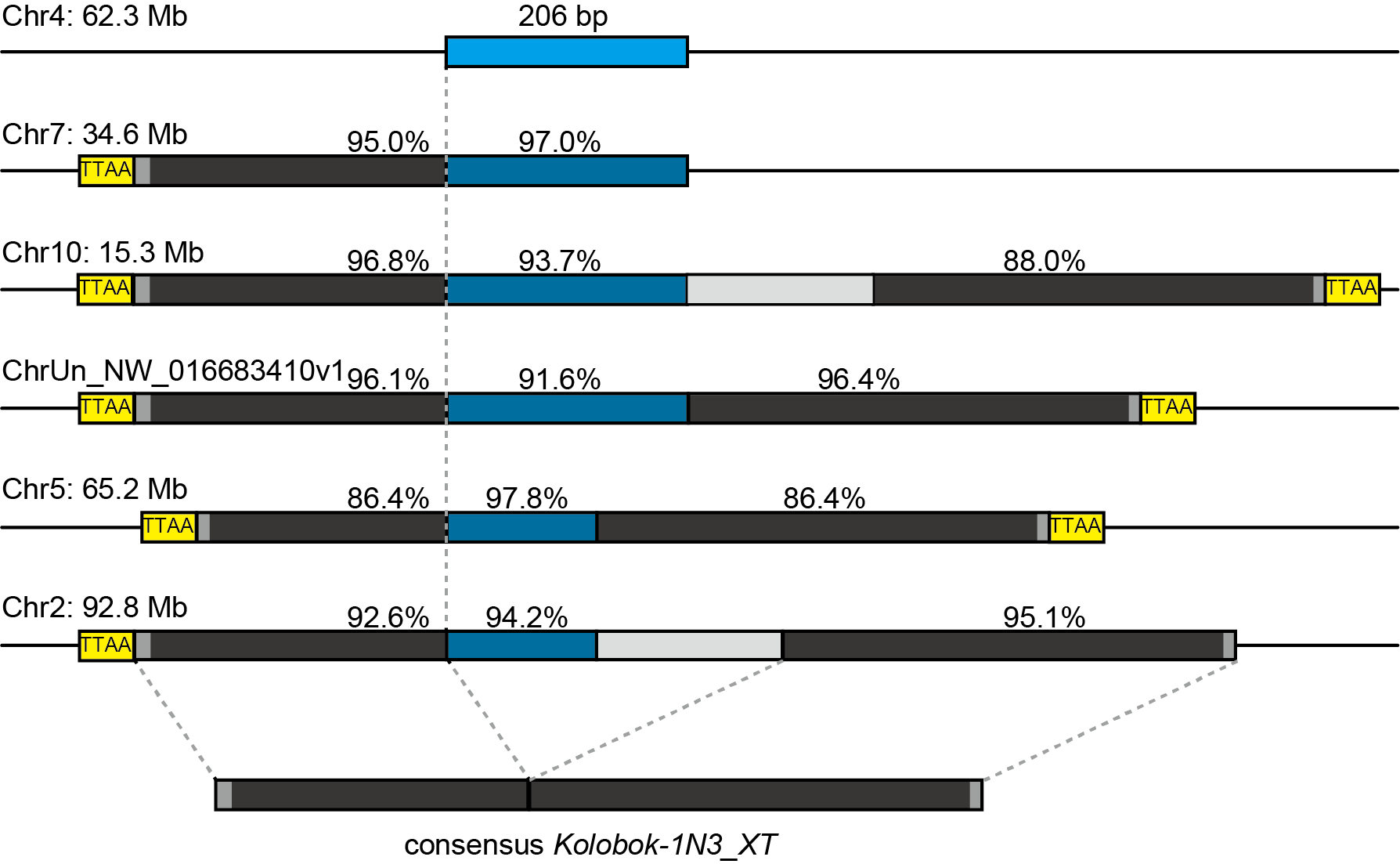


**Supplementary Figure 18. One demonstrative case in a western clawed frog associated with five transposition events.** The figure convention follows that of Fig. 1d. Frequent secondary deletions or duplications (light gray boxes) occurred and disrupted the canonical structure (internal sequences flanked by TIR TEs and TSDs) of Pack-TIRs.


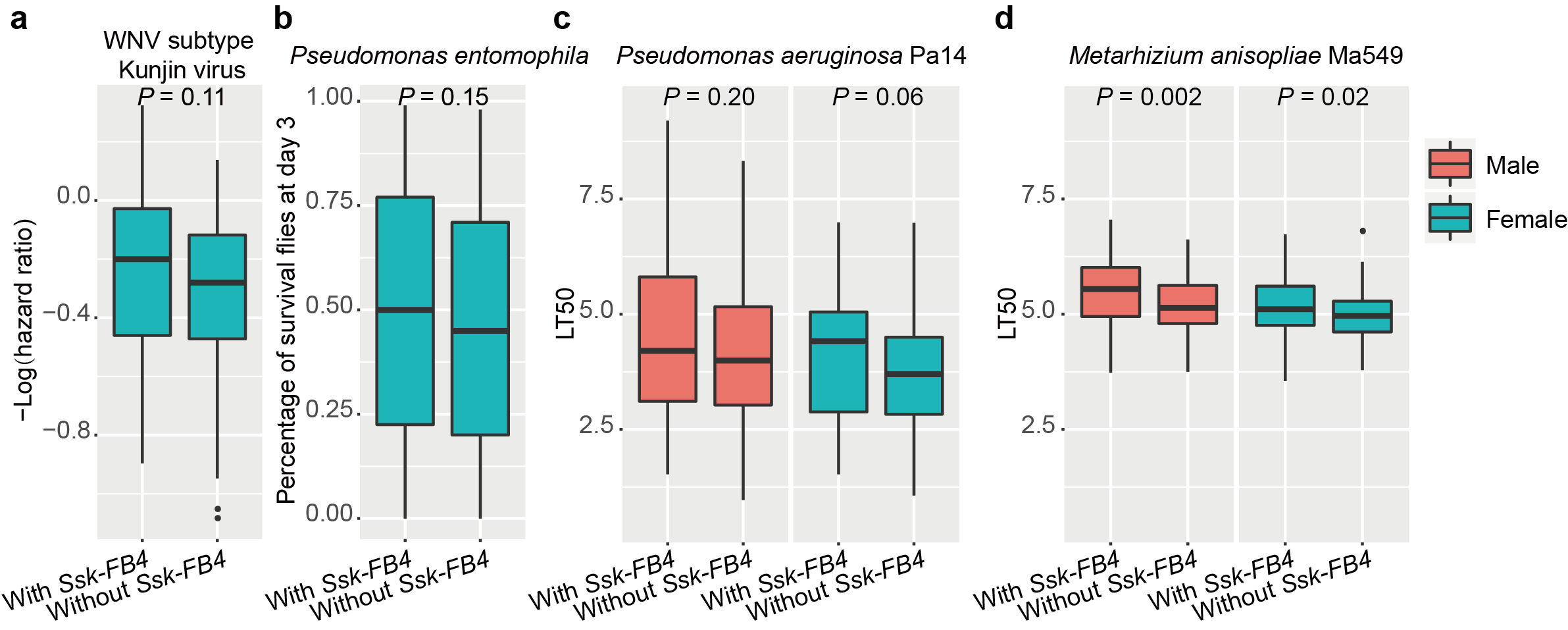


**Supplementary Figure 19. Survival status of DGRP lines infected with pathogens. a** DGRP lines infected with the WNV subtype Kunjin virus. Hazard ratio refers to the death rate of the infected group compared with control group (*n* = 34 samples for flies with *Ssk-FB4*, and *n* = 54 samples for flies without *Ssk-FB4*), and -log(hazard ratio) indicates the survival rate. **b** DGRP lines infected with the bacterium *Pseudomonas entomophila* (*n* = 47 samples for flies with *Ssk-FB4*, and *n* = 93 samples for flies without *Ssk-FB4*). **c** DGRP lines infected with the bacterium *Pseudomonas aeruginosa* Pa14 (*n* = 31 samples for male or female flies with *Ssk-FB4*, and *n* = 50 samples for male or female flies without *Ssk-FB4*). **d** Viability of DGRP lines infected with the fungus *Metarhizium anisopliae* Ma549 (*n* = 70 samples for male or female flies with *Ssk-FB4*, and *n* = 118 samples for male or female flies without *Ssk-FB4*). Box plots indicate the median (middle line), the interquartile range (IQR, box limits) with outliers beyond 1.5 times IQR. The one-sided Wilcoxon rank sum test was performed. For Panel c and d, the viability was quantified as LT50, *i.e.*, the days needed for half the flies to die.


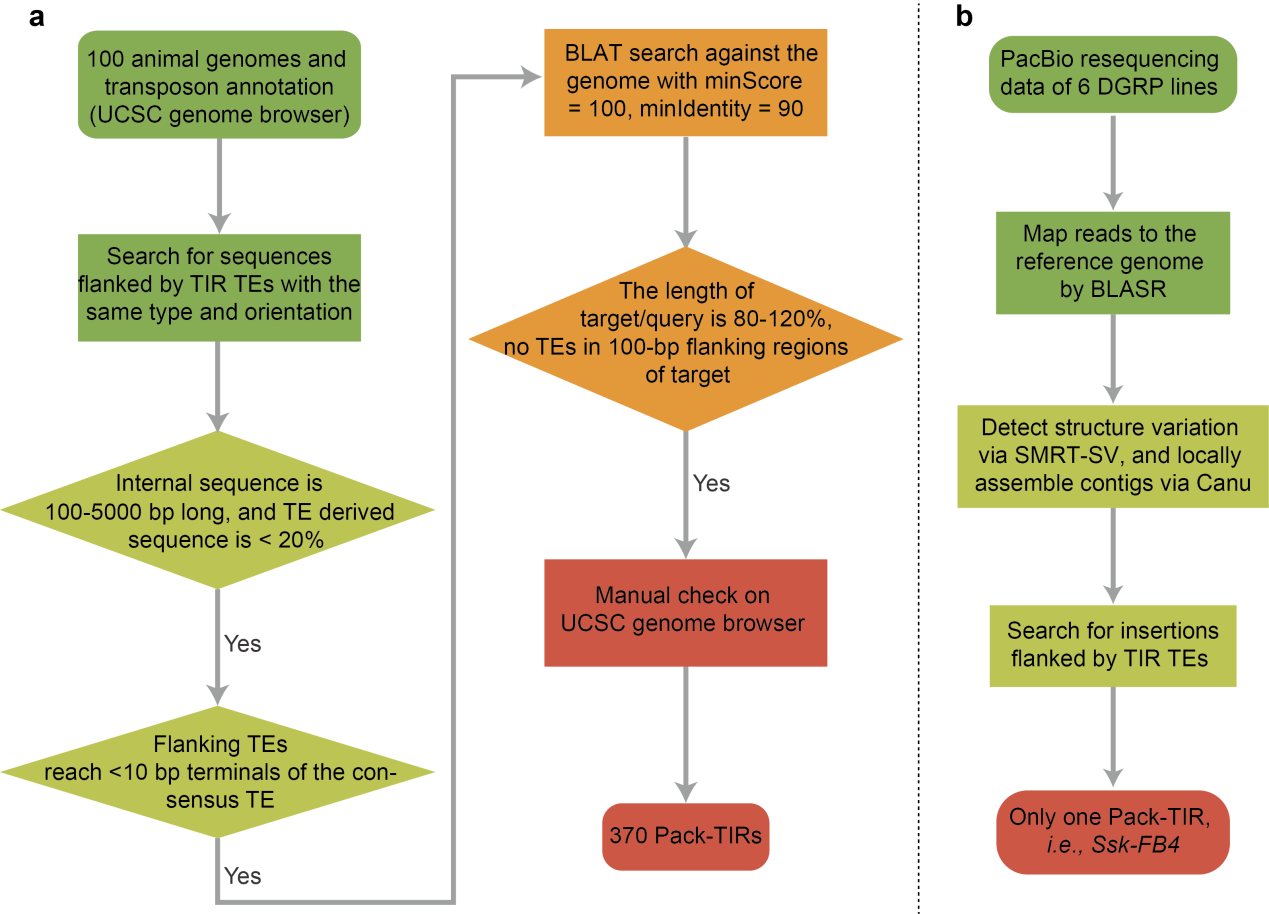


**Supplementary Figure 20. Workflow of Pack-TIR detection in 100 animal reference genomes (a) and population resequencing data of *D. melanogaster* (b).**

**Supplementary Tables**

| **Stage** | **Hi-C value** |
| --- | --- |
| Nuclear_cycle_12_whole_rep1 | -1.28 |
| Nuclear_cycle_12_whole_rep2 | 1.23 |
| Nuclear_cycle_14_anterior_rep1 | 0.32 |
| Nuclear_cycle_14_anterior_rep2 | -0.54 |
| Nuclear_cycle_14_whole_rep1 | -1.09 |
| Nuclear_cycle_14_whole_rep2 | -2.07 |
| Nuclear_cycle_14_posterior_rep1 | -0.72 |
| Nuclear_cycle_14_posterior_rep2 | 1.73 |
| Stage5_whole_rep1 | 5.75 |

**Supplementary Table 1. Interaction of *Ssk* and *FB4* of chr3L: 20.8 Mb during embryogenesis.** The Hi-C value refers to the normalized interaction intensity, which is defined as the intensity of the focal window (10 kb) minus the mean of the whole region including the focal window and four upstream or downstream windows (in total 90x90 kb) and then minus two-fold the standard deviation. Thus, a positive value means a stronger interaction relative to the flanking regions. Note: "stage 5" is roughly equivalent to the 14th nuclear cycle. Although these datasets do not give consistent results, positive values in four out of nine individual samples suggest a reasonable interaction strength between the two loci. In contrast, only one moderate interaction (0.40) out of nine samples could be observed for another *FB4* element (X: 22.5 Mb), and no interactions could be detected for the remaining four elements. Fig. 3D is based on the stage 5 sample due to its markedly higher sequencing depth and thus a more reliable signal.

| **Sample** | **Protein** | **Unique peptide** | **iBAQ value** |
| --- | --- | --- | --- |
| RAL-399 midgut rep1 | Ssk | GYMSDEGFLYVNSER | 4,638,866 |
| RAL-399 midgut rep2 | Ssk | GYMSDEGFLYVNSER | 3,760,395 |
| RAL-399 head rep1 | Ssk | GYMSDEGFLYVNSER | 44,606 |
| RAL-399 head rep2 | Ssk | GYMSDEGFLYVNSER | 44,387 |
| RAL-427 midgut rep1 | Ssk | GYMSDEGFLYVNSER | 3,117,661 |
| RAL-427 midgut rep2 | Ssk | GYMSDEGFLYVNSER | 3,229,940 |
| RAL-427 head rep1 | Ssk | GYMSDEGFLYVNSER | 16,636 |
| RAL-427 head rep2 | Ssk | GYMSDEGFLYVNSER | 16,827 |
| RAL-399 midgut rep1 | Ssk-FB4 chrX: 2.7 Mb | GHISDEGFLYVNSER | 22,939,865 |
|  |  | LINNLVIIFLYR |  |
| RAL-399 midgut rep2 | Ssk-FB4 chrX: 2.7 Mb | GHISDEGFLYVNSER | 23,312,284 |
|  |  | LINNLVIIFLYR |  |
| RAL-399 head rep1 | Ssk-FB4 chrX: 2.7 Mb | GHISDEGFLYVNSER | 144,372 |
| RAL-399 head rep2 | Ssk-FB4 chrX: 2.7 Mb | GHISDEGFLYVNSER | 142,219 |
| RAL-427 midgut rep1 | Ssk-FB4 chrX: 2.7 Mb | GHISDEGFLYVNSER | 4,554,948 |
| RAL-427 midgut rep2 | Ssk-FB4 chrX: 2.7 Mb | GHISDEGFLYVNSER | 5,104,326 |
| RAL-427 head rep1 | Ssk-FB4 chrX: 2.7 Mb | GHISDEGFLYVNSER | 36,136 |
| RAL-427 head rep2 | Ssk-FB4 chrX: 2.7 Mb | GHISDEGFLYVNSER | 21,861 |
| RAL-399 midgut Co-IP rep1 | Ssk | GYMSDEGFLYVNSER | 182,406 |
| RAL-399 midgut Co-IP rep2 | Ssk | GYMSDEGFLYVNSER | 513,086 |
| RAL-399 midgut Co-IP rep1 | Ssk-FB4 chrX: 2.7 Mb | GHISDEGFLYVNSER | 93,195 |
| RAL-399 midgut Co-IP rep2 | Ssk-FB4 chrX: 2.7 Mb | GHISDEGFLYVNSER | 460,837 |

**Supplementary Table 2. Intensity-based absolute quantification (iBAQ) value of Ssk and Ssk-FB4.** If there was more than one peptide, the sum of all intensities was used as the protein intensity. The protein intensities were further divided by the number of theoretically observable peptides as the final iBAQ values.

**Supplemental references**

1. Comeron, J.M., Ratnappan, R. & Bailin, S. The Many Landscapes of Recombination in *Drosophila melanogaster*. *PLOS Genetics* **8**, e1002905 (2012).
