# Supplementary material for: DNA transposons mediate duplications via transposition-independent and -dependent mechanisms in metazoans
Source: Nat Commun. 2021 Jul 13;12:4280. doi: 10.1038/s41467-021-24585-9 (PMC8277862; doi:10.1038/s41467-021-24585-9)
Supplement: Supplementary file 3 — Description of Additional Supplementary Files [file 41467_2021_24585_MOESM3_ESM.docx]

**Description for Supplementary Data files**

File Name: Supplementary Data 1

Description: **Three hundred seventy Pack-TIRs identified in animals.** Note: this list consists of four multicopy Pack-TIR families. #25, #358 and #359 (IDs in the first column) are actually multicopy, but their other members contain additional mutations (deletions or duplications in TEs) and thus show an identity lower than the cutoff in our pipeline (Methods). #341, #344 and #354 belong to one Pack-TIR family (Fig. 1D) and are all retained in our dataset. A large duplication containing Pack-TIR #296 generated #295 in the sloth. After excluding #341, #354, #295 and the redundant orthologous Pack-TIRs in primates (also see Supplementary Data 3), 281 cases were subsequently considered as nonredundant Pack-TIRs. The consensus TE is defined as that matching the consensus sequence in RepBase from start to end, which corresponds to "repStart=1 and repLeft=0" in RepeatMasker annotation files. The median divergence value was calculated between each individual copy and the consensus copy.

File Name: Supplementary Data 2

Description: **Number of Pack-TIRs and DNA TEs in each species.** The consensus TE was defined as that matching the consensus sequence in Repbase from start to end, which corresponds to "repStart=1 and repLeft=0" in RepeatMasker annotation files. In addition to the number of Pack-TIRs and total number of concensus TEs, the number of 16 individual superfamilies is also shown.

File Name: Supplementary Data 3

Description: **Orthology mapping and origination model for 97 nonredundant Pack-TIRs in primates.** The IDs follow those in Supplementary Data 1. Cases initially identified by our pipeline are marked in light blue. "+" or "-" represents the presence or absence of Pack-TIR across 18 primates (Columns Human to Bushbaby). If multiple IDs are shown in the first column, the corresponding cases are orthologous across the relevant species. For example, #160 and #209 are orthologous whereas their couterparts have been deleted in chimpanzees and bonobos. For another example, #127 was initially identified in gibbon, but emerged in the common ancestor of humans, chimpanzees, bonobos, gorillas, orangutans and gibbons. Because its orthologs did not pass the search cutoff (*e.g.*, TE insertion in the 100-bp flanking region of the parental copy), these were not identified in the initial search. For the Origination Model, 91 cases were compatible with post-transposition duplication (Fig. 2E) and were thus given the label "Gap-filling". For these 91 cases, we show only one species as an example (Column "Status of TE or Pack-TIR in the related species") which harbors TE but not the duplication (*e.g.*, #127), because other outgroup species show similar information. We could not make the inference for six cases (Supplementary Fig. 5) due to lack of information (*e.g.*, lacking orthologous TEs in the outgroup species) and thus labeled these as "Unknown". For five out of six cases (*e.g.*, #269), we show how the whole Pack-TIR together with its 5' and 3' flanking regions were absent in an example outgroup species. The last case ("#265, #178, #106, #288, #150, #162") is shared by 16 primates (lost in two species) suggesting an ancient origin.

File Name: Supplementary Data 4

Description: **Thirty-three Pack-TIRs in humans.** The 12 cases initially called by the pipeline are marked in blue. The features of the parental copy and Pack-TIRs are shown separately. The host genes refer to genes into which Pack-TIRs were duplicated.

File Name: Supplementary Data 5

Description: ***Ssk-FB4* loci among DGRP lines.** The individual *Ssk-FB4s* at chrX: 2.7 Mb, chr3R: 17.7 Mb and chr3R: 14.3 Mb were genotyped using split-read based method. The copy (chr3R: 17.7 Mb_TD) was genotyped based on the nucleotide changes relative to the copy at chr3R: 17.7 Mb. ST means standard karyotype, whereas INV stands for inversion (Huang *et al.*, 2014). Line 514 does not have inversion information and was thus labeled "NA". For four genotyping results, we relied on only one informatic read, as marked in the column "Additional information". Taking Row 26 as an example, most reads support the absence of *Ssk-FB4* with the exception of only one read supporting its presence. All other genotyping results are supported by more than one read.

File Name: Supplementary Data 6

Description: ***Ssk-FB4* loci identified in GDL lines.**

File Name: Supplementary Data 7

Description: **Presence or absence of the six *FB4s* in six DGRP lines sequenced by PacBio.** "+" indicates the presence of *FB4*, whereas "-" indicates the absence of *FB4*. Here, we also show the structural variations longer than 50 bp within each *FB4*.

File Name: Supplementary Data 8

Description: **Primers used to confirm and sequence *Ssk-FB4s* in five DGRP lines.** We designed three pairs of primers to sequence the full-length, upstream or downstream sequences of each *Ssk-FB4* copy. The numbers (bp) show the expected size. Full-length indicates that the two primers were located in the 5' and 3' flanking regions of *Ssk-FB4*. Upstream means that one primer was located in the 5' flanking region and the other was located inside *Ssk-FB4*. The downstream primers were defined analogously. The grids in colors represent three different *Ssk-FB4* loci detected by the split-read method, and all 15 presence/absence calls were validated by sequencing. The color code follows that in Fig. 3B.

File Name: Supplementary Data 9

Description: **Thirteen genome-wide association studies (GWAS) performed in DGRP lines.** The four datasets with sufficient statistical power were marked in blue. Reference in Row 15 and 16 are thesis rather than regular papers.
